# Determination of Organochlorine Pesticides in Green Leafy Vegetable Samples via Fe_3_O_4_ Magnetic Nanoparticles Modified QuEChERS Integrated to Dispersive Liquid-Liquid Microextraction Coupled with Gas Chromatography-Mass Spectrometry

**DOI:** 10.1155/2021/6622063

**Published:** 2021-03-13

**Authors:** Ling Yu, Guiquan Guo, Jun Zhao, Linnan Zhao, Aiqing Xia, Xu He, Cuijuan Xing, Lili Dong, Fang Wang

**Affiliations:** ^1^College of Chemistry and Chemical Engineering, Xingtai University, Xingtai 054001, China; ^2^Xingtai Department of Ecology and Environment, Xingtai 054001, China

## Abstract

A fast method based on Fe_3_O_4_ magnetic nanoparticles (Fe_3_O_4_ MNPs) modified QuEChERS integrated to dispersive liquid-liquid microextraction (DLLME) coupled with gas chromatography-mass spectrometry was established for the determination of 8 organochlorine pesticides (OCPs) in green leafy vegetables. The factors involved in the purification by QuEChERS and concentration by DLLME were optimized. In the QuEChERS process, Fe_3_O_4_ MNPs were used as a new impurity adsorbent after the sample extraction procedure by acetonitrile, which achieved phase separation rapidly. Carbon black was used as an alternative to costly graphitized carbon black without affecting the recovery. In the process of DLLME, 1 mL of the extract obtained by QuEChERS was used as the dispersive solvent, 40 *μ*L of chloroform was used as the extractive solvent, and 4 mL of water was added. Making them mix well, then the dispersed liquid-liquid microextraction concentration was subsequently carried out. The enrichment factors of 8 OCPs ranged from 22.8 to 36.6. The recoveries of the proposed method ranged from 78.6% to 107.7%, and the relative standard deviations were not more than 7.5%. The limits of detection and limits of quantification were 0.15–0.32 *μ*g/kg and 0.45–0.96 *μ*g/kg, respectively. The method has been successfully applied to the determination of OCPs in green leafy vegetable samples.

## 1. Introduction

Green leafy vegetables can provide essential nutrients such as vitamins, dietary fiber, and minerals, which have been deemed as essential in people's daily life. However, a large number of insect pests growing with the vegetables lead to the overuse of pesticides and insecticides, resulting in serious damage to the environment. Organochlorine pesticides (OCPs) are one kind of excellent synthetic chlorinated organic compound that has been used worldwide due to their low cost and high insecticidal efficiency. While the OCPs are highly neurotoxic and have the potential to bioaccumulate on fatty tissues and biomagnification through the food chain, suppressing the human's immune system, in the meantime, the highly toxic and nonbiodegradable OCPs would cause huge damage to the ecological environment [1, 2]. Hence, OCPs have been banned in developed countries since the mid-70s. Although nearly 40 years have passed, these substances are still of concern. Due to their high stability, long half-life, difficult degradation, and slow metabolism in organisms, the detection rates of OCPs in environmental media [3, 4] and food [5, 6] are still very high. With people's craving for healthy food, the safety of vegetables has attracted more and more attention. Therefore, it is necessary to develop sensitive and selective methods to monitor OCPs in vegetables.

OCPs are polychlorinated compounds, which are highly electronegative elements and have the characteristics of high-temperature gasification. They are usually separated by gas chromatography (GC) and then qualitatively/quantitatively detected by electron capture detector (ECD) [7–9] or mass spectrometry (MS) [10–12]. Among them, gas chromatography-mass spectrometry (GC-MS) exhibits excellent selectivity and sensitivity. It adopts full SCAN mode for qualitative analysis and selected ion monitor (SIM) mode for quantitative analysis, which is widely used in the detection of low-concentration organic compounds.

In the field of pesticide residue analysis, traditional sample pretreatment technologies include solid-phase extraction [13, 14] and liquid-liquid extraction [15, 16]. These traditional pretreatment methods are cumbersome and time-consuming, with large consumption of organic reagents and poor selectivity for trace analysis. With the development of sample pretreatment technology, miniaturized and economical sample pretreatment technologies have been applied to the detection of pesticide residues in vegetables. Recently, researchers have developed fast, sensitive, and environmentally friendly pretreatment methods to separate and enrich pesticide residues in different matrixes such as solid-phase microextraction (SPME) [17], gel permeation chromatography (GPC) [18], QuEChERS [19], and DLLME [20]. The matrix of green leafy vegetables is complex, which contains pigment, organic acid, cellulose, and so on. In order to determine trace levels of pesticide residues accurately, purification and enrichment processes are required. Theoretically, the combination of QuEChERS and DLLME can achieve a good purification effect and high enrichment.

QuEChERS was firstly proposed by professor Anastassiadas of the United States Department of Agriculture in 2003 [21]. This method is to extract samples, conduct dispersed solid-phase extraction with various purifiers, and finally separate pesticides from the sample matrix by centrifugation. QuEChERS is simple and fast and has a wide range of detection. It has been widely used in the analysis of pesticide residues in fish [22], meat [23], dairy products [24], tea [25], fruits, and vegetables [26]. However, the traditional QuEChERS technology still requires multistep centrifugation separation, which seriously affects the pretreatment speed of batch samples and has the disadvantages of low enrichment multiple and high detection limit. Fe_3_O_4_ MNPs have received much attention because of their low cost, high surface area, rapid isolation, and short time for extraction from a sample solution with a strong magnet [27, 28]. Fe_3_O_4_ MNPs can effectively adsorb impurities in vegetables, reduce detection background, and increase detection sensitivity. Moreover, external magnets can be used for phase separation, which avoids time-consuming centrifugal separation and makes extraction simpler and more efficient. Therefore, Fe_3_O_4_ MNPs combined with QuEChERS have been studied to rapidly purify the sample matrix [29, 30]. However, QuEChERS is not suitable for the determination of low-concentration samples because of its high detection limit and low enrichment factor. DLLME is a sample pretreatment technology integrating sampling, extraction, and concentration first proposed by Rezaee et al. in 2006 [31]. The advantages of DLLME are simple operation, high enrichment efficiency, and low amount of organic solvent, which is particularly suitable for application in the field of trace analysis. However, DLLME has the defects of poor selectivity and poor purification ability. Therefore, the combination of Fe_3_O_4_ MNPs modified QuEChERS with DLLME shows the advantages of fast separation of Fe_3_O_4_ MNPs, strong purification of QuEChERS, and high enrichment ability of DLLME. The proposed method will be helpful for better extraction and quantitative analysis of pesticide residues in green leafy vegetables and provide a reference for future research and routine monitoring of pesticide residues.

In this work, Fe_3_O_4_ MNPs modified QuEChERS integrated to DLLME coupled with GC-MS was used to determine 8 OCPs in green leafy vegetables. Moreover, systematical optimization was studied in detail during the various influencing factors of QuEChERS and DLLME. The method is simple, fast, safe, and cheap with good repeatability. This may be the first report about the application of Fe_3_O_4_ MNPs-QuEChERS-DLLME-GC-MS for the analysis of pesticides in real samples.

## 2. Experimental

### 2.1. Instruments and Reagents

Shimadzu GC/MS-QP 2010 Ultra (Shimadzu Corporation, Japan). SC-3612 centrifuge from Zhongkezhongjia Scientific Instrument Co. Ltd. (Anhui, China), KQ-50E ultrasonic device from Kunshan ultrasonic instrument Co. Ltd. (Kunshan, China), and a DS-1 blender from Shanghai specimen model factory (Shanghai, China) were used in the sample treatment.

OCP standards (50 *μ*g/mL) of *α*-, *β*-, *γ*-, and *δ*-hexachlorocyclohexanes (HCH), p,p′-DDE ([2,2-bis(p-chlorophenyl)-1,1-dichloroethylene]), p,p′-DDD(dichlorodiphenyldichloro-ethane), o,p′-DDT ([1,1,1-trichloro-2,2-bis-(p-chlorophenyl) ethane]), and p,p′-DDT ([1,1,1- trichloro-2,2-bis-(p-chlorophenyl) ethane]) were purchased from China Institute of metrology (Beijing, China). Primary secondary amine (PSA) with an average particle diameter of 40–60 *μ*m was obtained from Weiqiboxing Biotechnology Co. Ltd. (Wuhan, China). Carbon black bondesil with a particle size of 40–60 *μ*m was purchased from Biosun (Japan). Fe_3_O_4_ MNPs were obtained from Aladdin (Shanghai, China). HPLC-grade methanol was from Fisher Scientific (New Jersey, USA). Acetonitrile (ACN), acetone, sodium chloride (NaCl), anhydrous magnesium sulfate (MgSO_4_), dichloromethane, carbon tetrachloride, chloroform, and chlorobenzene were purchased from Yongda Chemical Reagent Co. Ltd. (Tianjin, China). Ultrapure water was produced using a purification system MilliQ Direct Q (Millipore, Brazil).

The mixed standard solution at 10 *μ*g/mL was prepared in HPLC-grade methanol and stored at 4°C. The working standard solutions were prepared as follows.

The 10 *μ*g/mL OCPs mixed standard stock solution was diluted into series of standard working solutions of 0.02, 0.05, 0.1, 0.2, 0.4, 0.6, 0.8, and 1.0 *μ*g/mL with HPLC-grade methanol to calculate the recovery after QuEChERS.

The 10 *μ*g/mL OCPs mixed standard stock solution was diluted into series of standard working solutions of 0.25, 0.5, 1.0, 2.0, and 4.0 *μ*g/mL with chloroform, which is used to calculate recovery and enrichment factor after DLLME.

The mixed standard stock solution was added to the blank vegetable matrix. The standard addition levels were 5, 10, 20, 50, and 100 *μ*g/kg. The QuEChERS and DLLME procedures were then performed.

### 2.2. GC-MS Conditions

The GC was fitted with a Rxi-5si1MS column (30 m × 0.25 mm inner diameter × 0.25 *μ*m film thickness, Shimadzu, USA). Helium was used as the carrier gas at a flow rate of 1 mL/min. The inlet temperature was 250°C. The injected volume was 1 *μ*L (working in the splitless mode). The oven temperature program was set as follows: 50°C as initial temperature, maintained for 1 min, raised to 200°C at 20°C/min, raised to 230°C at 5°C/min, and maintained for 5 min, raised to 280°C at 10°C/min, and held for 1 min. The total run time was 25.5 min. The mass spectrometer was operated in electron impact ionization mode at 70 eV. The ionization source temperature was 260 °C with a solvent delay time of 5 min. The interface temperature was 280°C. Initially, the MS detector was operated in the scan mode (from 40 to 500 *m*/*z*) to select target ions. Subsequently, all samples were analyzed in SIM mode; at least three specific ions were selected and sorted into groups. Retention time and quantitative and qualitative ions for pesticides are given in Table 1.

### 2.3. Sample Preparation

The four types of green leafy vegetables (spinach, cabbage, oilseed rape, and lettuce) were purchased from local supermarkets (Xingtai, China). Samples were chopped and homogenized and stored at −18°C in glass containers. Subsequently, the sample pretreatment method was adopted in the following steps.

#### 2.3.1. QuEChERS Step

Two g of the homogenized vegetable sample was transferred into a 10 mL glass centrifuge tube. Four mL of ACN was added, and the tube was hand-shaken for 1 min to ensure that the solvent interacted well with the matrix. Next, 0.8 g of NaCl was added, and this solution was also shaken for 1 min to prevent salt agglomeration. After that, the mixture was centrifuged at 3500 rpm for 3 min. Then, the upper ACN extract was collected for purification. Take 2 mL ACN extract into a 10 mL glass centrifuge tube. Add 10 mg PSA, 20 mg carbon black, and 20 mg Fe_3_O_4_ MNPs successively to the tube. The mixture was ultrasonicated for 3 min. Then, an external magnetic field was used for the separation of the adsorbents from the solution.

#### 2.3.2. DLLME Step

In this step, 4 mL of ultrapure water was added into 1 mL solution (ACN phase) obtained from the previous step. The mixture was rapidly injected into 40 *μ*L chloroform (extraction solvent). The obtained mixed liquid was shaked vigorously and then ultrasonicated for 1 min; and the analytes were extracted into the extraction solvent droplets. The solution was centrifuged for 3 min at 3500 rpm which led to the settlement of the dispersed droplets of the organic phase at the bottom of the tube. The sedimentary facies were taken into a 1.5 mL tip bottom centrifugal tube. A small amount of MgSO_4_ was added to remove the trace water in the sedimentary facies. Finally, 1 *μ*L of the sedimentary facies was injected into the GC-MS system for analysis.

## 3. Results and Discussion

### 3.1. Optimization of the QuEChERS Step

#### 3.1.1. Optimization of Extraction Solvent

**Figure 1:**
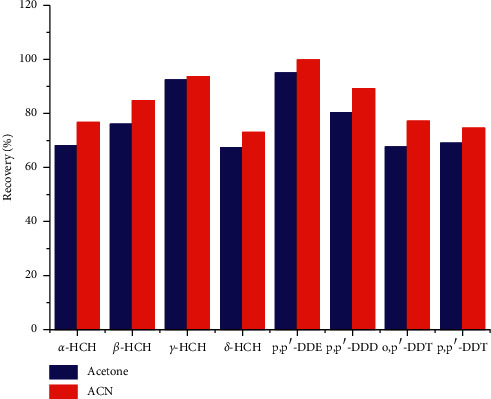
Extraction efficiency of two different organic solvents for OCPs.

The study obtained from the blank vegetable matrix spiked at the same concentration (100 *μ*g/kg of OCPs) was calculated. An extraction agent should extract the analytes from the samples and also acts as a suitable disperser solvent in the following DLLME step. Moreover, the miscibility of the dispersive solvent in both the extraction solvent and aqueous phase plays a vital role in the subsequent DLLME step. Methanol, acetone, and ACN endowed excellent miscibility in both extraction solvent and aqueous phase. Herein, they were used as an extraction solvent in this study. The results showed that when methanol was used as the extraction agent, the solution was not stratified after adding NaCl, and the organic phase could not be obtained; then, the follow-up experiments cannot be carried out smoothly. However, acetone and ACN exhibited better extraction performance on OCPs, which ranged from 67.6% to 97.8% by acetone and 74.5% to 99.8% by ACN as shown in Figure 1. In the meantime, ACN has stronger selectivity to OCPs and the organic phase can be separated from water quickly through salting out. Therefore, ACN was chosen as a suitable extraction agent.

#### 3.1.2. Selection of Purification Agent

Green leafy vegetables contain water, sugar, fatty acid, protein, pigment, and so forth. In order to reduce the matrix effect, it is necessary to further purify the sample extract. PSA can effectively remove fatty acids, sugars, and proteins from the matrix. The traditional QuEChERS method uses graphitized carbon black (GCB) to adsorb pigments, but GCB is relatively expensive. In this study, carbon black is used as a purifying agent, which could offer significant cost-saving with retaining OCPs and removing pigments.

In China the price of GCB is 900 yuan for 5 g. It costs 1.8 yuan to make 10 mg for each sample while the price of carbon black is significantly reduced which is only 95 yuan for 500 g, namely, 0.0019 yuan per 10 mg. It shows an obvious price advantage for batch processing detection. Moreover, according to [32], PSA, C18, and GCB were used as sorbent, and the recoveries of OCPs ranged from 66% to 111% while, in this study, PSA, carbon black, and Fe_3_O_4_ MNPs were used as sorbent, and the recoveries of OCPs ranged from 62% to 102%. Both GCB and black carbon have similar recoveries of OCPs and pigment removal. On the other hand, Fe_3_O_4_ MNPs have the advantages of fast separation and pigment removal. Thus, PSA, carbon black, and Fe_3_O_4_ MNPs were chosen as the purification agent for the further steps.

#### 3.1.3. Optimization of the Sorbent Weight

**Figure 2:**
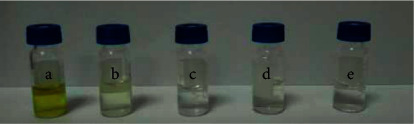
Different amounts of black carbon removal effect: 0 mg (a), 10 mg (b), 20 mg (c), 40 mg (d), and 60 mg (e).

**Figure 3:**
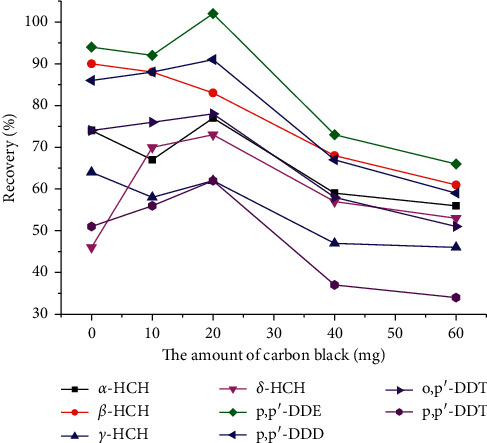
Effect of different amounts of carbon black on recoveries of OCPs.

The recoveries of analytes at different weights of PSA, carbon black, and Fe_3_O_4_ MNPs were investigated at the same concentration (0.2 *μ*g/mL of OCPs) in vegetable matrix extract. Firstly, the amount of PSA was increased from 0 mg to 40 mg in the condition of fixing the amount of carbon black and Fe_3_O_4_ MNPs. The results indicate that, with the increase of PSA dosage, recoveries of OCPs firstly increased and then decreased. Relatively high analytical signals were obtained when PSA dosage was 10 mg. Secondly, different weights of Fe_3_O_4_ MNPs (10, 20, 40, and 80 mg) were investigated while keeping the amount of PSA and carbon black unchanged. The results reveal that when Fe_3_O_4_ MNPs were 10 mg, it is difficult to rapidly separate the sorbent from the solution through external magnetic field attraction, which might be because that the number of Fe_3_O_4_ MNPs was few and the generated magnetism was too small. But when Fe_3_O_4_ MNPs were increased to 20 mg, the recoveries of OCPs were better and rapid separation can be achieved by magnets. When Fe_3_O_4_ MNPs increased to 40 and 60 mg, the recoveries began to decrease, which was due to the adsorption of OCPs by the increased Fe_3_O_4_ MNPs. Finally, the purification effect of carbon black at 0, 10, 20, 40, and 60 mg was investigated with a fixed amount of PSA and Fe_3_O_4_ MNPs, and the optimum content of carbon black was determined; the results are shown in Figures 2 and 3.  Figure 2 demonstrates that the optimum amount of carbon black was 20 mg; all colors of the solution were removed.  Figure 3 shows the recoveries of OCPs ranging from 61.8% to 102.3%, reaching the optimal level. With the increase of black carbon, the recoveries of OCPs began to decline. Hence, the optimum contents of PSA, Fe_3_O_4_ MNPs, and carbon black were 10 mg, 20 mg, and 20 mg, respectively.

#### 3.1.4. Study of Ultrasonic Time

**Figure 4:**
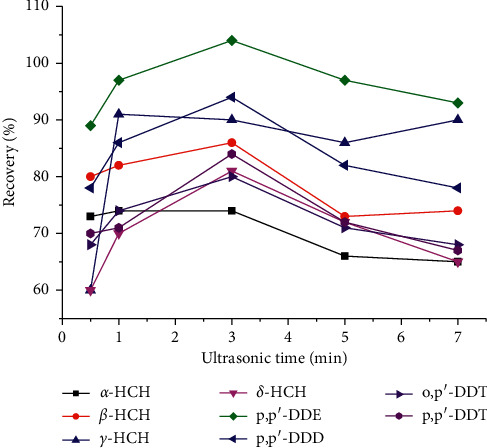
Selection of ultrasonic time.

Ultrasonic time is a critical parameter in partitioning the impurities between the solution and the sorbent. To obtain a suitable ultrasonic time, 0.5, 1, 3, 5, and 7 min were tested and the results are shown in Figure 4. It reveals that recoveries of 8 OCPs except for the *α*-HCH and *γ*-HCH increased up to the maximum (with the values ranging from 73.9% to 104.1%) at 3 min. Thus, 3 min was chosen as the ultrasonic time for the following steps.

### 3.2. Optimization of DLLME Conditions

In order to improve the efficiency and quickly optimize the experimental parameters, the mixed standard solution was diluted to 0.025 *μ*g/mL with the blank matrix purification solution after QuEChERS, and DLLME experiment condition optimization was carried out. Extraction efficiency under different conditions was evaluated by extraction recovery (ER) and enrichment factor (EF). The calculation formulas of ER and EF are as follows:

(1) $$\begin{array}{c} \text{ER}\%=\frac{C_{\text{sed}}\times V_{\text{sed}}}{C_{0}\times V_{0}}\times 100,\\ \text{EF}=\frac{C_{\text{sed}}}{C_{0}}, \end{array}$$

where *C*_sed_ is the concentration of the target in the sedimentary facies, *C*_0_ is the concentration of ACN purification solution (0.025 *μ*g/mL), *V*_sed_ is the volume of the sedimentary facies, and *V*_0_ is the volume of ACN purification solution (1 mL).

#### 3.2.1. Selection of Extraction Solvents and Volumes

**Figure 5:**
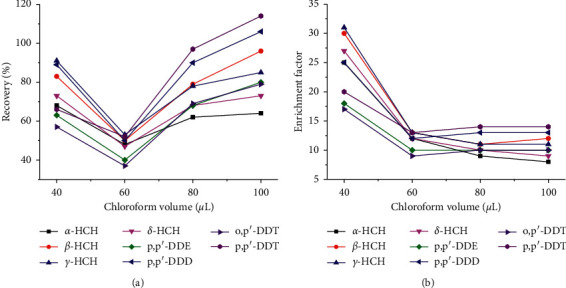
Effect of chloroform volume on OCPs recoveries (a) and enrichment factors (b).

ACN was used as the extraction agent in the above QuEChERS; thus, it was also used as the dispersant in DLLME. And 1 mL of each sample was purified with QUECHERS for DLLME; only the type and volume of extractant need to be considered. Extraction solvents play the main role in all DLLME procedures to achieve high ERs. Herein, the effects of carbon tetrachloride, chloroform, chlorobenzene, and dichloromethane on the extraction of 8 OCPs were investigated. The results showed no sedimentary facies when dichloromethane was used as an extraction agent. When chlorobenzene was used as an extraction agent, there were more impurity peaks in the chromatogram. When carbon tetrachloride was used as an extraction agent, the ERs were low, ranging from 38.8% to 60.0%. Chloroform has a good selectivity to the target peak and good recoveries of 53.8–95.7%. Therefore, chloroform was selected as the optimal extraction solvent in further studies. Also, the influence of chloroform volume on the extraction efficiency was studied. For this purpose, different volumes of chloroform (20, 40, 60, 80, and 100 *μ*L) were tested. The results showed that when chloroform was 20 *μ*L, there were no sedimentary facies after DLLME. By increasing the volume of chloroform from 40 to 100 *μ*L, ERs increased firstly and then decreased (Figure 5(a)) and EFs decreased to 60 *μ*L and then remained constant (Figure 5(b)). Notably, when the volume of chloroform was 40 *μ*L, ERs were 61.9–98.4% and EFs were 16.7–30.7. However, when the volume increased to 60 *μ*L, ERs and EFs decreased and reached the lowest, which were 47.4–61.1% and 9.3–13.3, respectively.

When the volume further increased to 80 *μ*L and 100 *μ*L, the ERs increased again with the values of 62.4–97.0% and 64.0–114.1% respectively. However, the EFs ranged from 8.9 to 13.9 and 8.0 to 14.3, respectively. Given the comprehensive effect of ERs and EFs, 40 *μ*L was selected as the optimum volume of extraction solvent in the following steps.

#### 3.2.2. Selection of Water Volume

QuEChERS was used for sample purification, and DLLME was used for the enrichment of target components in samples. However, DLLME must be implemented in a water environment by adding extraction agent and dispersant. Therefore, a certain volume of water should be added to the ACN extract after QuEChERS in the early stage to create a water environment for DLLME. For finding the suitable water volume, 2, 3, 4, 5, and 6 mL were examined. When the water volume was 2 mL, there were no sedimentary facies. It could be attributed to the small water volume, resulting in complete dissolution of dispersant (ACN), extractant (chloroform), and water. With the increase of the water volume, the extractant was evenly dispersed in the aqueous phase, forming an emulsion system of water/dispersant/extractant. After centrifugation, stable sedimentary facies were formed. It is noteworthy that when the volume of water was 4 mL, the ERs of 8 OCPs in the sedimentary facies reached the optimal level from 65.5% to 103.2% while the ERs gradually decreased when the volume of water was larger than 5 mL. It could be explained as follows: because the proportion of dispersant (ACN) in the system decreased with the increase of water, the extraction agent (chloroform) was not evenly dispersed in the aqueous phase, resulting in the reduced recovery. Thus, 4 mL was selected as the optimum volume of water in the following steps.

#### 3.2.3. Selection of Ultrasonic Time

Effects of different extraction times (0.5, 1, 3, 5, and 7 min) on ERs of 8 OCPs were investigated under ultrasound irradiation. The results presented that ERs increased from 0.5 min to 1 min but decreased from 1 min to 7 min. The highest ERs were 72.9–109.4% at 1 min. With the increase in the dispersion of extraction solvent (chloroform) in the solution by ultrasonic irradiation, the ERs of targets were improved. However, if the ultrasonic time is too long, a lot of heat will be generated. The heat will increase the volatility of the solvent and reduce the extraction efficiency. Thus, 1 min was chosen as the optimum time in this work.

### 3.3. Validation of Method

**Figure 6:**
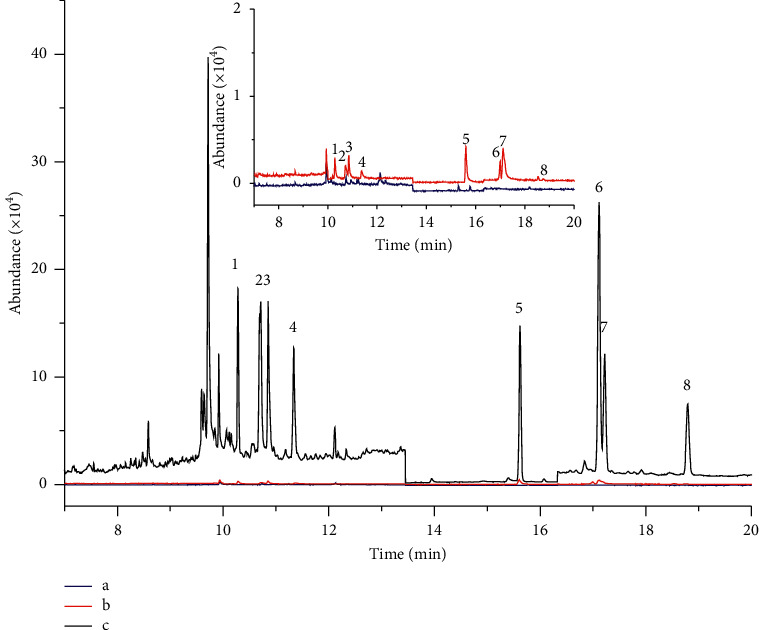
Typical GC-MS chromatograms of (a) blank matrix of spinach sample in SIM mode using the optimized QuEChERS procedure. (b) A standard spiked with 0.025 *μ*g/mL of 8 OCPs in the blank matrix purification solution after QuEChERS. (c) A standard spiked with 0.025 *μ*g/mL of 8 OCPs in the blank matrix purification solution after QuEChERS and then DLLME. (1) *α*-HCH, (2) *β*-HCH, (3) *γ*-HCH, (4) *δ*-HCH, (5) p,p′-DDE, (6) p,p′-DDD, (7) o,p′-DDT, and (8) p,p′-DDT.

Validation experiments were carried out by using pesticide-free vegetable samples spiked with 8 OCPs mixture standard solution at series of concentrations (1, 10, 20, 50, 80, and 100 *μ*g/kg). Validation of the present method was carried out following the sample pretreatment method. The samples were purified by QuEChERS and enriched by DLLME. Under the optimized conditions above, standard curves were plotted to target quantitation ion peak area (*y*) to the corresponding concentration (*x*). Different parameters such as linear range, linear equation, limit of detection (LOD), quantification (LOQ), and enrichment factor were determined (see Table 2). Results indicate that the linear range was 1–100 *μ*g/kg with the correlation coefficients (*R*^2^) obtained ranging from 0.9954 to 0.9992. The LODs and LOQs were estimated as three and ten times the signal-to-noise (S/N) ratio, respectively. LODs ranged from 0.15 to 0.32 *μ*g/kg and LOQs ranged from 0.45 to 0.96 *μ*g/kg. These results ensured that the method had a high sensitivity, repeatability, and a wide linear range. Sample pretreatment under optimal conditions showed a good purification effect, the chromatogram of the blank matrix was very clean, and a small amount of impurity peak had no interference to the target (Figure 6(a)). By comparing Figures 6(b) and 6(c), it could be concluded that the enrichment effect was obvious after DLLME, and the enrichment multiple of 8 OCPs was between 22.8 and 36.6.

Recoveries and repeatability were provided by recovery experiments of spiked OCPs in different vegetable matrices at three different concentration levels (2, 20, and 40 *μ*g/kg) with five replicates each. The obtained results are listed in Table 3. As shown in Table 3, the recoveries of 8 OCPs ranged from 78.6% to 107.7% and relative standard deviations (RSDs) were not more than 7.5%, which indicated that the method proposed in this study was of great accuracy, repeatability, and application value for the determination of OCPs in green leafy vegetables.

### 3.4. Comparison with Other Methods

Considering that DLLME is generally used for simple matrix samples (such as water), but not used for complex substrates such as vegetables, therefore, the performance of the Fe_3_O_4_ MNPs-QuEChERS-DLLME method was compared with other methods (QuEChERS [32] and QuEChERS-DLLME [33]) from the viewpoint of the amount of extract, purification dosage, amount of organic reagent in the concentration process, pretreatment time, recovery, and LOD; the data are given in Table 4. Notably, this method has shown a shorter pretreatment time compared with the other methods; that is, Fe_3_O_4_ MNPs modified QuEChERS enabled impurity to be completely isolated from sample solutions in a short time by using an external magnetic field. Additionally, it has the advantage of cost-saving for using low-cost carbon black instead of GCB.

### 3.5. Analysis of Real Samples

The method established in this paper was applied to detect vegetable samples. Four kinds of green leafy vegetables (spinach, cabbage, oilseed rape, and lettuce) obtained were analyzed in three replicates. The results showed that the concentrations of all analytes were lower than their LOQs consistent with the literature reported by the literature studied by Qiu et al. in 2018 [33].

## 4. Conclusion

A new method was proposed combining Fe_3_O_4_ MNPs modified QuEChERS-DLLME for the purification and preconcentration of OCPs from green leafy vegetables. ACN was used as an extraction agent in QuEChERS, and the impurities were extracted out of ACN by adsorbents. Then, the ACN phase was the disperser solvent in the DLLME step. The magnetic separation greatly improved the speed of phase separations, which saved analysis time and did not decrease the cleanup efficiency. The substitution of carbon black for GCB exhibits a better removal performance of the pigment and cost-saving. Moreover, this method obtained good recoveries (78.6–107.7%) and repeatability (RSD ≤7.5%) as well as lower LODs (0.15–0.32 *μ*g/kg) and LOQs (0.45–0.96 *μ*g/kg) and enhanced EFs (22.8–36.6). This study provides a reference for the determination of OCPs in green leafy vegetables.

## Figures and Tables

**Table 1:** Retention time and quantitative and qualitative ions of 8 OCPs.

Peak number	Compound	Retention time (min)	Mass-to-charge ratio (*m*/*z*)	
			Quantitative ion	Qualitative ion
1	*α*-HCH	10.265	181	181, 183, 219
2	*β*-HCH	10.665	109	109, 181, 183
3	*γ*-HCH	10.830	181	181, 183, 219
4	*δ*-HCH	11.310	181	181, 183, 219
5	p,p′-DDE	15.585	246	246, 248, 318
6	p,p′-DDD	17.080	235	235, 237, 165
7	o,p′-DDT	17.190	235	235, 237, 165
8	p,p′-DDT	18.750	235	235, 237, 165

**Table 2:** Linear range, linear equation, LOD, LOQ, and enrichment factor of 8 OCPs.

Organochlorine	Linearity range (*μ*g/kg)	Linear equation	Coefficient of association (*r*)	LOD (*μ*g/kg)	LOQ (*μ*g/kg)	Enrichment factor
*α*-HCH	1–100	*y* = 2028*x* − 2280.6	0.9986	0.22	0.66	27.4
*β*-HCH	1–100	*y* = 1628.1*x* − 3347.9	0.9969	0.21	0.63	33.1
*γ*-HCH	1–100	*y* = 1413.5*x* + 6447.2	0.9987	0.23	0.69	36.6
*δ*-HCH	1–100	*y* = 1270.8 *x* − 2302	0.9992	0.31	0.93	29.3
p,p′-DDE	1–100	*y* = 1885.6*x* + 2662.5	0.9992	0.15	0.45	25.2
p,p′-DDD	1–100	*y* = 6429.7 *x* − 12109	0.9988	0.24	0.72	35.8
o,p′-DDT	1–100	*y* = 3444.5 *x* − 6867.5	0.9990	0.22	0.66	22.8
p,p′-DDT	1–100	*y* = 1802.4 *x* − 1684.5	0.9988	0.32	0.96	26.6

**Table 3:** Recoveries (*n* = 5) and RSDs obtained from 8 OCPs spiked in spinach, cabbage, oilseed rape, and lettuce at three levels.

Compounds	Spiked (*μ*g/kg)	Spinach		Cabbage		Oilseed rape		Lettuce	
		Recovery (%)	RSD (%)	Recovery (%)	RSD (%)	Recovery (%)	RSD (%)	Recovery (%)	RSD (%)
*α*-HCH	2	88.7	3.1	98.7	3.5	89.3	1.2	84.4	6.4
	20	101.2	3.2	98.0	5.5	85.1	2.7	88.3	5.4
	40	92.0	4.5	103.2	4.3	78.6	3.8	80.1	4.7

*β*-HCH	2	106.5	6.6	104.7	7.0	95.5	7.4	98.5	2.9
	20	89.0	4.3	92.3	5.4	91.6	2.7	96.3	5.8
	40	103.1	7.2	89.9	4.9	88.5	5.1	102.0	5.3

*γ*-HCH	2	92.3	3.8	97.1	2.1	94.7	1.6	101.4	1.8
	20	88.9	4.9	98.4	1.7	104.3	3.7	103.2	1.1
	40	97.2	3.3	101.3	2.8	96.6	4.1	94.7	4.2

*δ*-HCH	2	99.9	2.4	95.4	2.5	95.6	7.3	85.5	5.1
	20	96.5	3.5	100.2	5.7	90.2	5.7	80.6	3.6
	40	107.7	4.7	105.7	6.0	102.9	4.2	79.0	4.6

p,p′-DDE	2	102.4	4.6	88.6	3.7	106.5	3.6	100.8	1.8
	20	105.3	6.7	87.1	2.2	96.6	2.0	102.6	2.6
	40	97.0	5.9	90.5	4.3	90.8	2.1	91.7	3.7

p,p′-DDD	2	96.0	6.8	91.6	3.7	89.3	5.6	95.6	7.5
	20	92.7	4.5	92.3	2.1	85.4	4.8	95.2	5.4
	40	95.5	7.5	82.0	3.2	98.5	1.7	102.3	4.6

o,p′-DDT	2	87.2	4.5	84.2	2.4	89.9	2.2	94.8	1.6
	20	98.3	3.2	88.9	1.6	96.6	1.5	104.7	3.2
	40	96.8	2.4	90.8	3.1	102.1	3.4	89.3	3.1

p,p′-DDT	2	87.3	1.6	78.7	2.9	95.0	2.5	87.0	2.4
	20	85.5	2.5	80.6	3.5	91.5	1.4	96.3	4.5
	40	95.1	5.3	85.0	4.7	92.3	4.0	92.5	2.5

**Table 4:** Comparison of this method with other literature.

Pretreatment method	Amount of extract required for 1 g sample	Purification dosage required for 1 mL extract	Concentration method	Amount of organic reagent in the concentration process	Instrument	Pretreatment time (min)	Recovery (%)	LOD (*μ*g/kg)	Literature
QuEChERS	2 mL ACN 1.2 g MgSO_4_, 0.3 g NaCl, 0.15 g Na2HCit·1.5H2O 0.3 g Na3Cit·2H2O	QuEChERS:33 mg PSA, 100 mg MgSO_4_ 33 mg C18 33 mg GCB	Nitrogen stream	1 mL Hexane	GC-ECD	16–17	66–111	*α*-HCH: 0.93 HCB: 3.33 *β*-HCH: 1.71 Lindane: 0.94 *δ*-HCH: 1.42 Aldrin: 2.09 *α*-Endosulfan: 1.77 p,p′-DDE: 2.26 Dieldrin: 2.26 Endrin: 2.08 *β*-Endosulfan: 2.93 p,p′-DDD: 3.38 o,p′-DDT: 3.16 Methoxychlor: 1.41	[32]
QuEChERS-DLLME	2 mL ACN 0.8 g MgSO_4_ 0.2 g NaCl	QuEChERS: 50 mg PSA	DLLME	40 *μ*L undecanol	GC-ECD	19–20	77.2–112.5	*α*-BHC: 1.02 *β*-BHC: 1.13 *γ*-BHC: 0.93 *δ*-BHC: 0.75 Heptachlor: 1.04 Aldrin: 1.28 Heptachlor Epoxide: 0.45 *α*-Chlordane: 1.33 Endrin: 0.64 *β*-Endosulfan: 1.17 p,p′-DDD: 1.32 Endrin aldehyde: 0.96 Endrin ketone: 1.25	[33]
Fe_3_O_4_ MNPs-QuEChERS-DLLME	2 mL ACN0.4 g NaCl	QuEChERS: 10 mg carbon black 10 mg Fe_3_O_4_ MNPs	DLLME	40 *μ*L chloroform	GC-MS	12–13	78.6–107.7	*α*-HCH: 0.22 *β*-HCH: 0.21 *γ*-HCH: 0.23 *δ*-HCH: 0.31 p,p′-DDE: 0.15 p,p′-DDD: 0.24 o,p′-DDT: 0.22 p,p′-DDT: 0.32	This study

## Data Availability

The data used to support the findings of this study are included within the article.

## Supplementary Materials

Supplementary Materials Figure S1: effect of different amount of PSA on OCPs recoveries. Figure S2: effect of different amount of Fe3O4 MNPs on OCPs recoveries.
