# Supplementary material for: Determination of Organochlorine Pesticides in Green Leafy Vegetable Samples via Fe3O4 Magnetic Nanoparticles Modified QuEChERS Integrated to Dispersive Liquid-Liquid Microextraction Coupled with Gas Chromatography-Mass Spectrometry
Source: J Anal Methods Chem. 2021 Mar 13;2021:6622063. doi: 10.1155/2021/6622063 (PMC7994098; doi:10.1155/2021/6622063)
Supplement: Supplementary Materials — Figure S1: effect of different amount of PSA on OCPs recoveries. Figure S2: effect of different amount of Fe3O4 MNPs on OCPs recoveries. [file 6622063.f1.doc]

Supporting Information

**Determination of Organochlorine Pesticides in Green Leafy** **Vegetable Samples via Fe_3_O_4_ Magnetic Nanoparticles Modified QuEChERS Integrated to** **Dispersive Liquid-Liquid Microextraction Coupled with** **Gas Chromatography-Mass Spectrometry**

Ling Yu,^1*^Guiquan Guo,^1*^ Jun Zhao,^1^ Nan Lin,^2^ Aiqing Xia,^1^ Xu He,^1^ Cuijuan Xing,^1^ Lili Dong,^1^ and Fang Wang^1^

^1^ College of Chemistry and Chemical Engineering, Xingtai University, Xingtai 054001, China

^2^ Environmental Monitoring Center of Xingtai, Xingtai 054001, China

*3.1.3 Optimization of the sorbent weight.* The recoveries of analytes at different weight of PSA, carbon black and Fe_3_O_4_ MNPs were investigated at the same concentration (0.2 μg/mL of OCPs) in vegetable matrix extract. Firstly, the amount of PSA was increased from 0 mg to 40 mg in the condition of fixing the amount of carbon black and Fe_3_O_4_ MNPs. The results indicate that with the increase of PSA dosage, recoveries of OCPs firstly increased and then decreased. Relatively high analytical signals were obtained when PSA dosage was 10 mg (FIGURE S1). Secondly, different weights of Fe_3_O_4_ MNPs (10, 20, 40 and 80 mg) were investigated while keeping the amount of PSA and carbon black unchanged. The results reveal that when Fe_3_O_4_ MNPs was 10 mg, it is difficult to rapidly separate the purifier from the solution through external magnetic field attraction, which might because that the amount of Fe_3_O_4_ MNPs was few and the generated magnetism was too small. But When Fe_3_O_4_ MNPs was increased to 20 mg, the recoveries of OCPs were better and rapid separation can be achieved by magnets（FIGURE S2）. When Fe_3_O_4_ MNPs increased to 40 and 60 mg, the recoveries began to decrease, which was due to the adsorption of OCPs by the increased Fe_3_O_4_ MNPs.


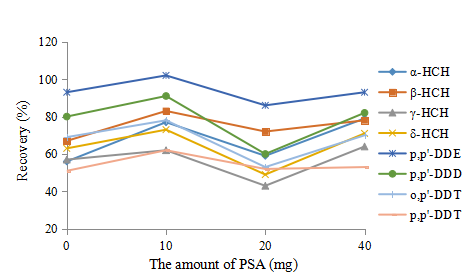


FIGURE S1 Effect of different amount of PSA on OCPs recoveries


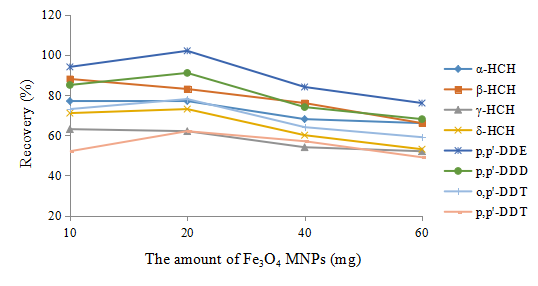


FIGURE S2 Effect of different amount of Fe_3_O_4_ MNPs on OCPs recoveries
